# Peer Review of “A Local Community-Based Social Network for Mental Health and Well-being (Quokka): Exploratory Feasibility Study”

**DOI:** 10.2196/33930

**Published:** 2021-10-27

**Authors:** Ashwag Alasmari

**Affiliations:** 1 University of Maryland Baltimore County Baltimore, MD United States

**Keywords:** local social network, community health, well-being, digital health, consumer health

*This is a peer-review report submitted for the paper “A Local Community-Based Social Network for Mental Health and Well-being (Quokka): Exploratory Feasibility Study.”*

## Round 1 Review

This research [1] conducted an exploratory study of local community-based interventions and evaluated the intervention’s potential for promotion of local, social, and unfamiliar activities as they pertain to healthy habits. I like how the authors built the motivation of this research. The introduction is well written and a good list of background information is provided. I am not sure what Quokka is, if it already exists or was just developed for the purpose of the study. I would like to see some background information on this application. I also would appreciate if the authors looked at their findings through the lens of prior work. How similar or different is this research to prior work?

## References

[1] Shih C, Pudipeddi R, Uthayakumar A, Washington P (2021). A local community-based social network for mental health and well-being (Quokka): exploratory feasibility study. JMIRx Med.

